# Supplementary material for: The L27 Domain of MPP7 enhances TAZ-YY1 Cooperation to Renew Muscle Stem Cells
Source: bioRxiv. 2023 Nov 4:2023.11.01.565166. Preprint. [Version 1] doi: 10.1101/2023.11.01.565166 (PMC10635061; doi:10.1101/2023.11.01.565166)
Supplement: Supplement 1 — Figure S1. Additional data to support Figure 1. (A) Scheme for FACS isolation of YFP-marked Con and Mpp7 cKO MuSCs from hindlimb muscles; FACS profiles to the right. (B) Percentages of MPP7+ MuSCs from Con and Mpp7 cKO immediately after FACS isolation. (C) Representative IF images of PAX7 and LAMININ for Con and Mpp7 cKO TA muscles 30 d (days) after TMX regimen without injury; white arrows, PAX7+ MuSCs. Quantification of PAX7+ SCs is to the right. N = 3 mice, each. (D, E) Representative images of H&E histology (D) and IF (E) at 21 dpi. Black arrows indicate regenerated myofibers, and dashed lines, injury boundary in (D); white arrows, PAX7+ cells in (E); associated with Figures 1G and 1H. (F) Representative images of EdU Click-reaction with IF of YFP; yellow arrowheads, EdU+YFP+ cells; white arrows, EdU−YFP+ cells; associated with Figure 1I. (G) Representative IF images of PAX7 and MYOD with DAPI from single myofiber cultures; asterisks, PAX7+ cells; white arrows, PAX7+MYOD+ cells; yellow arrows, MYOD+ cells; associated with Figure 1J. (H-J) Regimen to determine EdU incorporation and programmed cell death of MuSCs in culture in (H). (I, J) Percentages of EdU+ and cleaved-Caspase 3+ cells, respectively. DOXO was used to demonstrate cleaved-Caspase 3 reactivity. Data information: Scale bars = 25 μm in (C-F, J). Error bars represent means ± SD. Student’s t-test (two-sided). n.s, P > 0.05; ***, P < 0.001. Figure S2. Additional data to support Figure 2. (A) IF of M-Cadherin (MCAD), N-Cadherin (NCAD), β-Catenin (CTNNB1), and PAR3 in Con and Mpp7 cKO MuSCs immediately after single myofiber isolation; yellow arrowheads, apical side. Quantified fluorescent signals (AU) are to the right; total 30 cells in each group. N = 2 Con mice; N = 3 Mpp7 cKO mice. (B) Distribution pattern of V5-MPP7 variants in transfected Mpp7 cKO MuSCs on single myofibers was determined by IF of V5; yellow arrowheads, apical side. Relative fractions of nuclear versus non-nuclear MPP7 are to the right: [file media-1.pdf]

Fig. S1

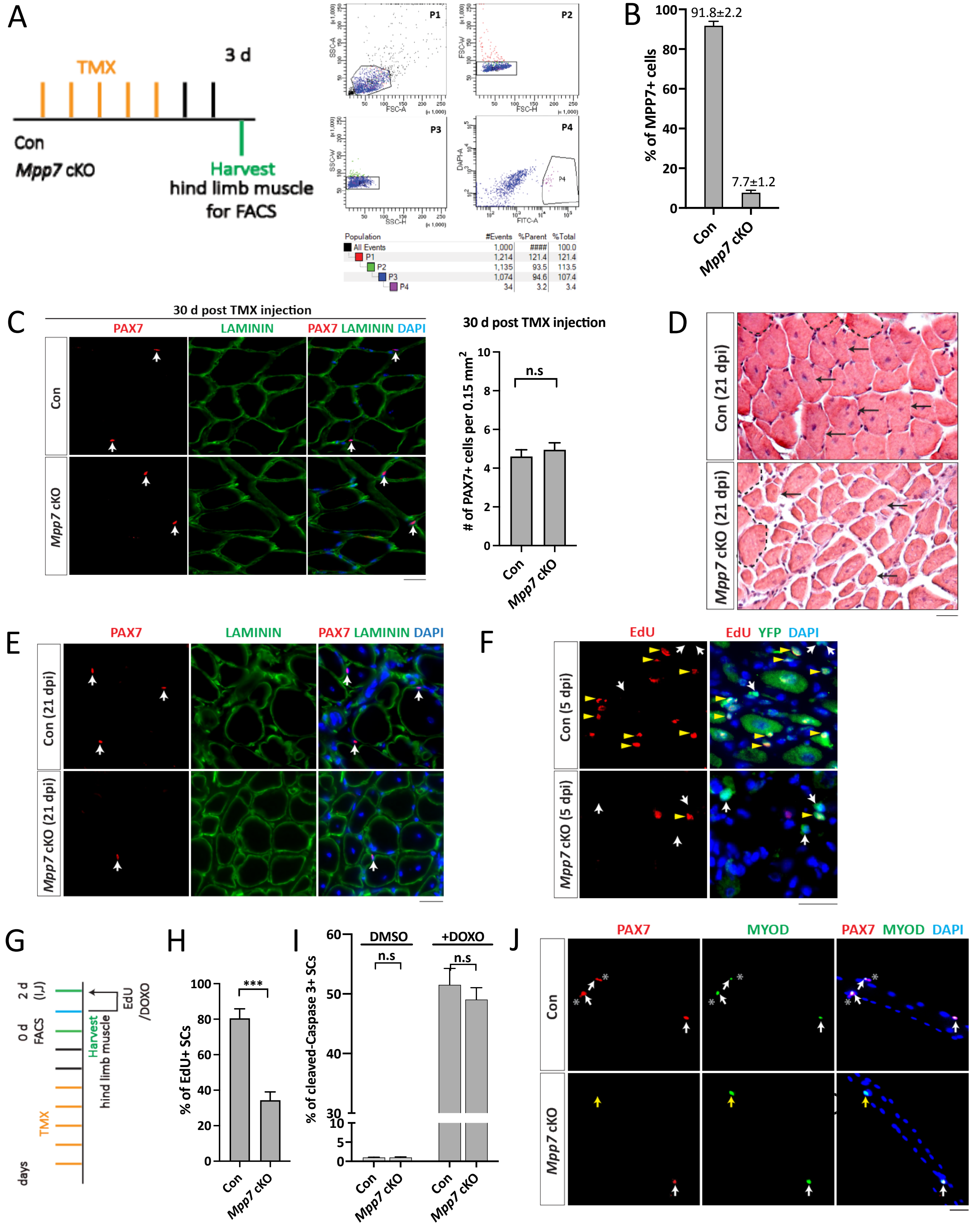

A
TMX
3 d
Con
Mpp7 cKO
Harvest
hind limb muscle
for FACS
P1
P2
P3
P4
Population
All Events
B
100
91.8±2.2
% of MPP7+ cells
7.7±1.2
Con
Mpp7 cKO
C
30 d post TMX injection
PAX7
LAMININ
PAX7 LAMININ DAPI
# of PAX7+ cells per 0.15 mm2
n.s
D
Con (21 dpi)
Mpp7 cKO (21 dpi)
E
F
EdU
EdU YFP DAPI
Con (5 dpi)
Mpp7 cKO (5 dpi)
G
days
TMX
Harvest
hind limb muscle
FACS
EdU
/DOXO
0 d
2 d
(I,J)
H
% of EdU+ SCs
***
I
DMSO
+DOXO
% of cleaved-Caspase 3+ SCs
J
MYOD
PAX7 MYOD DAPI

Fig. S2

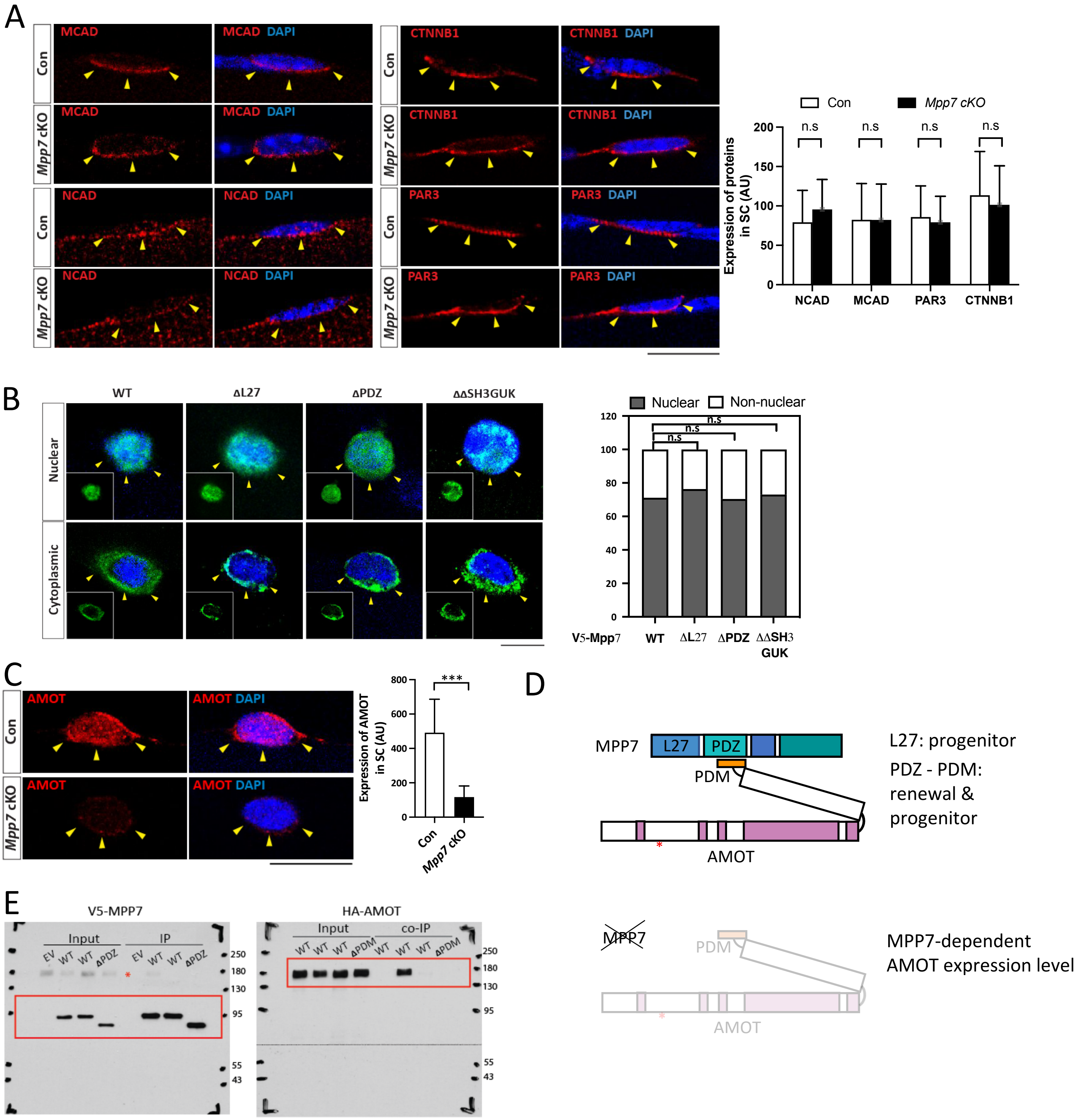

A
MCAD
MCAD DAPI
NCAD
NCAD DAPI
CTNNB1
CTNNB1 DAPI
PAR3
PAR3 DAPI
Con
Mpp7 cKO
Expression of proteins in SC (AU)
n.s
NCAD
MCAD
PAR3
CTNNB1
B
WT
ΔL27
ΔPDZ
ΔΔSH3GUK
Nuclear
Cytoplasmic
Non-nuclear
V5-Mpp7
C
AMOT
AMOT DAPI
Expression of AMOT in SC (AU)
***
D
MPP7
L27
PDZ
PDM
AMOT
L27: progenitor
PDZ - PDM: renewal & progenitor
MPP7-dependent AMOT expression level
E
V5-MPP7
HA-AMOT
Input
IP
co-IP
EV
WT
ΔPDZ
ΔPDM

Fig. S3

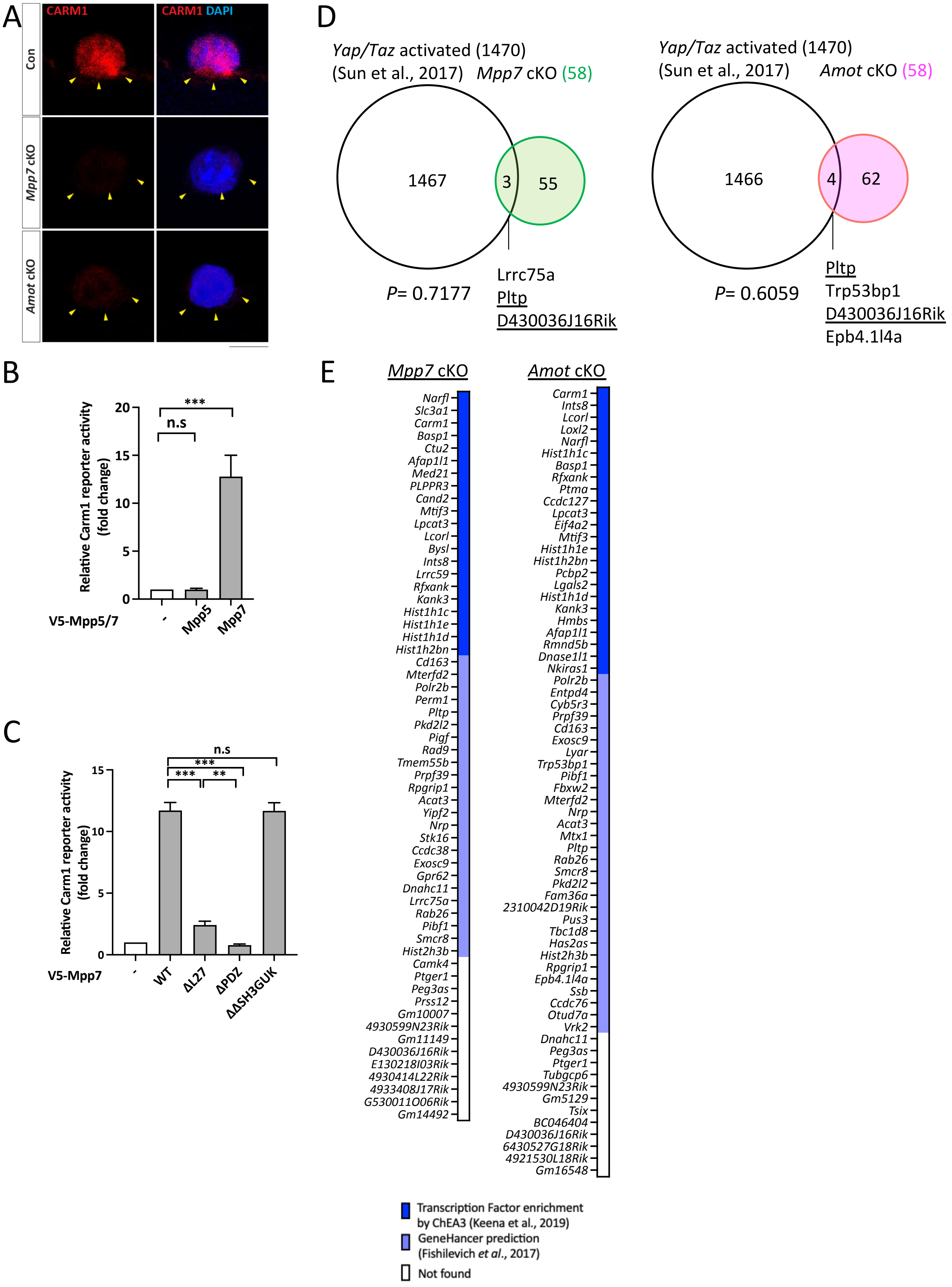

A
CARM1
CARM1 DAPI
Con
Mpp7 cKO
Amot cKO
B
Relative Carm1 reporter activity (fold change)
20
15
10
5
0
***
n.s
V5-Mpp5/7
-
Mpp5
Mpp7
C
Relative Carm1 reporter activity (fold change)
15
10
5
0
n.s
***
***
**
V5-Mpp7
-
WT
ΔL27
ΔPDZ
ΔΔSH3GUK
D
Yap/Taz activated (1470)
(Sun et al., 2017)
Mpp7 cKO (58)
1467
3
55
P= 0.7177
Lrrc75a
Pltp
D430036J16Rik
Yap/Taz activated (1470)
(Sun et al., 2017)
Amot cKO (58)
1466
4
62
P= 0.6059
Pltp
Trp53bp1
D430036J16Rik
Epb4.1l4a
E
Mpp7 cKO
Narfl
Slc3a1
Carm1
Basp1
Ctu2
Afap1l1
Med21
PLPPR3
Cand2
Mtif3
Lpcat3
Lcorl
Bysl
Ints8
Lrrc59
Rfxank
Kank3
Hist1h1c
Hist1h1e
Hist1h1d
Hist1h2bn
Cd163
Mterfd2
Polr2b
Perm1
Pltp
Pkd2l2
Pigf
Rad9
Tmem55b
Prpf39
Rpgrip1
Acat3
Yipf2
Nrp
Stk16
Ccdc38
Exosc9
Gpr62
Dnahc11
Lrrc75a
Rab26
Pibf1
Smcr8
Hist2h3b
Camk4
Ptger1
Peg3as
Prss12
Gm10007
4930599N23Rik
Gm11149
D430036J16Rik
E130218I03Rik
4930414L22Rik
4933408J17Rik
G530011O06Rik
Gm14492
Amot cKO
Carm1
Ints8
Lcorl
Loxl2
Narfl
Hist1h1c
Basp1
Rfxank
Ptma
Ccdc127
Lpcat3
Eif4a2
Mtif3
Hist1h1e
Hist1h2bn
Pcbp2
Lgals2
Hist1h1d
Kank3
Hmbs
Afap1l1
Rmnd5b
Dnase1l1
Nkiras1
Polr2b
Entpd4
Cyb5r3
Prpf39
Cd163
Exosc9
Lyar
Trp53bp1
Pibf1
Fbxw2
Mterfd2
Nrp
Acat3
Mtx1
Pltp
Rab26
Smcr8
Pkd2l2
Fam36a
2310042D19Rik
Pus3
Tbc1d8
Has2as
Hist2h3b
Rpgrip1
Epb4.1l4a
Ssb
Ccdc76
Otud7a
Vrk2
Dnahc11
Peg3as
Ptger1
Tubgcp6
4930599N23Rik
Gm5129
Tsix
BC046404
D430036J16Rik
6430527G18Rik
4921530L18Rik
Gm16548
Transcription Factor enrichment by ChEA3 (Keena et al., 2019)
GeneHancer prediction (Fishilevich et al., 2017)
Not found

Fig. S4

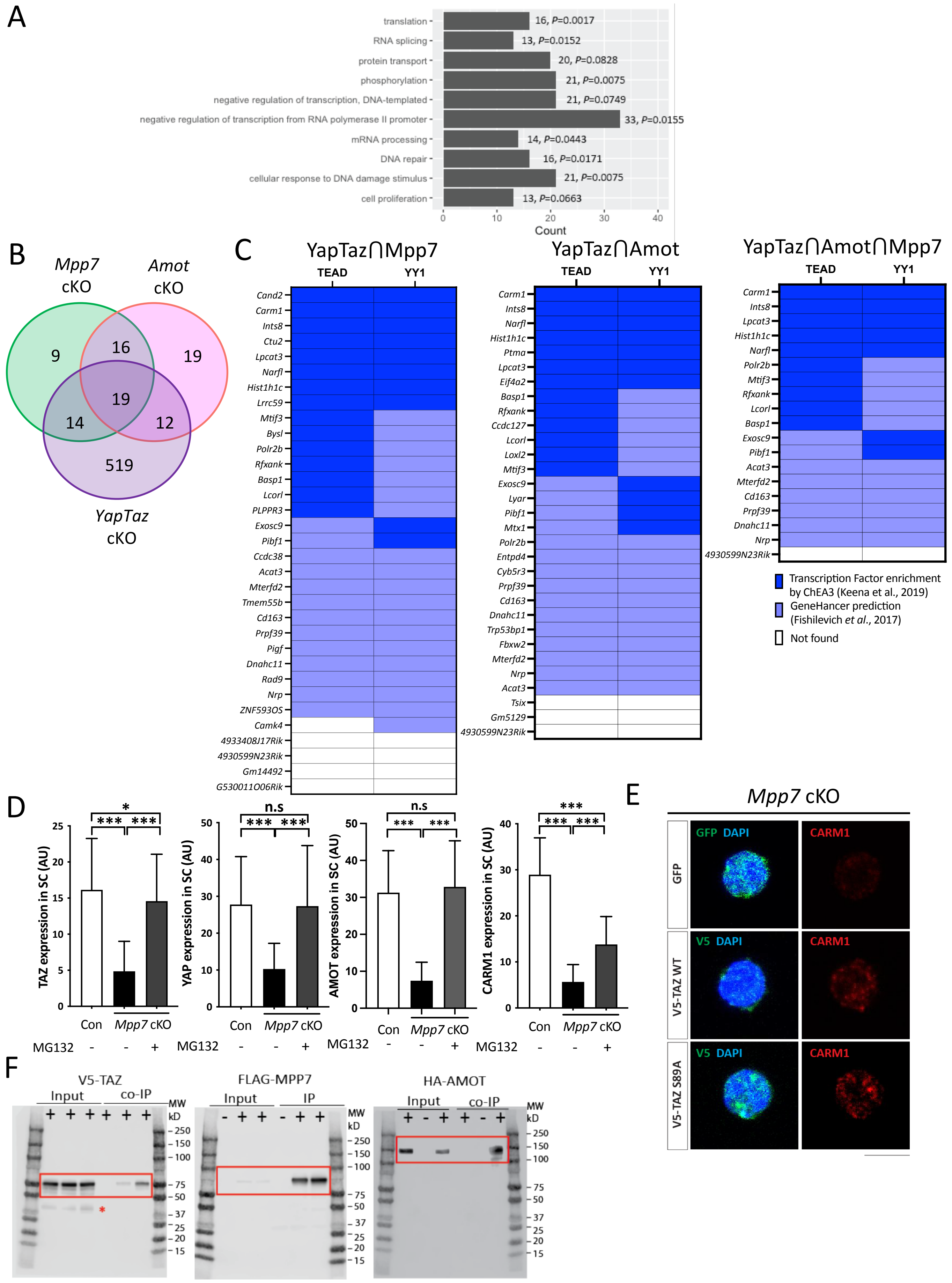

A
translation
RNA splicing
protein transport
phosphorylation
negative regulation of transcription, DNA-templated
negative regulation of transcription from RNA polymerase II promoter
mRNA processing
DNA repair
cellular response to DNA damage stimulus
cell proliferation
16, P=0.0017
13, P=0.0152
20, P=0.0828
21, P=0.0075
21, P=0.0749
33, P=0.0155
14, P=0.0443
16, P=0.0171
21, P=0.0075
13, P=0.0663
0 10 20 30 40
Count
B
Mpp7 cKO
Amot cKO
YapTaz cKO
9
16
19
19
14
12
519
C
YapTaz∩Mpp7
TEAD YY1
Cand2
Carm1
Ints8
Ctu2
Lpcat3
Narfl
Hist1h1c
Lrrc59
Mtif3
Bysl
Polr2b
Rfxank
Basp1
Lcorl
PLPPR3
Exosc9
Pibf1
Ccdc38
Acat3
Mterfd2
Tmem55b
Cd163
Prpf39
Pigf
Dnahc11
Rad9
Nrp
ZNF593OS
Camk4
4933408J17Rik
4930599N23Rik
Gm14492
G530011O06Rik
YapTaz∩Amot
TEAD YY1
Carm1
Ints8
Narfl
Hist1h1c
Ptma
Lpcat3
Eif4a2
Basp1
Rfxank
Ccdc127
Lcorl
Loxl2
Mtif3
Exosc9
Lyar
Pibf1
Mtx1
Polr2b
Entpd4
Cyb5r3
Prpf39
Cd163
Dnahc11
Trp53bp1
Fbxw2
Mterfd2
Nrp
Acat3
Tsix
Gm5129
4930599N23Rik
YapTaz∩Amot∩Mpp7
TEAD YY1
Carm1
Ints8
Lpcat3
Hist1h1c
Narfl
Polr2b
Mtif3
Rfxank
Lcorl
Basp1
Exosc9
Pibf1
Acat3
Mterfd2
Cd163
Prpf39
Dnahc11
Nrp
4930599N23Rik
Transcription Factor enrichment by ChEA3 (Keena et al., 2019)
GeneHancer prediction (Fishilevich et al., 2017)
Not found
D
TAZ expression in SC (AU)
YAP expression in SC (AU)
AMOT expression in SC (AU)
CARM1 expression in SC (AU)
*
n.s
n.s
***
Con
Mpp7 cKO
MG132 - - +
E
Mpp7 cKO
GFP
V5-TAZ WT
V5-TAZ S89A
GFP DAPI
V5 DAPI
CARM1
F
V5-TAZ
Input co-IP
FLAG-MPP7
Input IP
HA-AMOT
Input co-IP
MW kD
250
150
100
75
50
37
25
20
15

Fig. S5

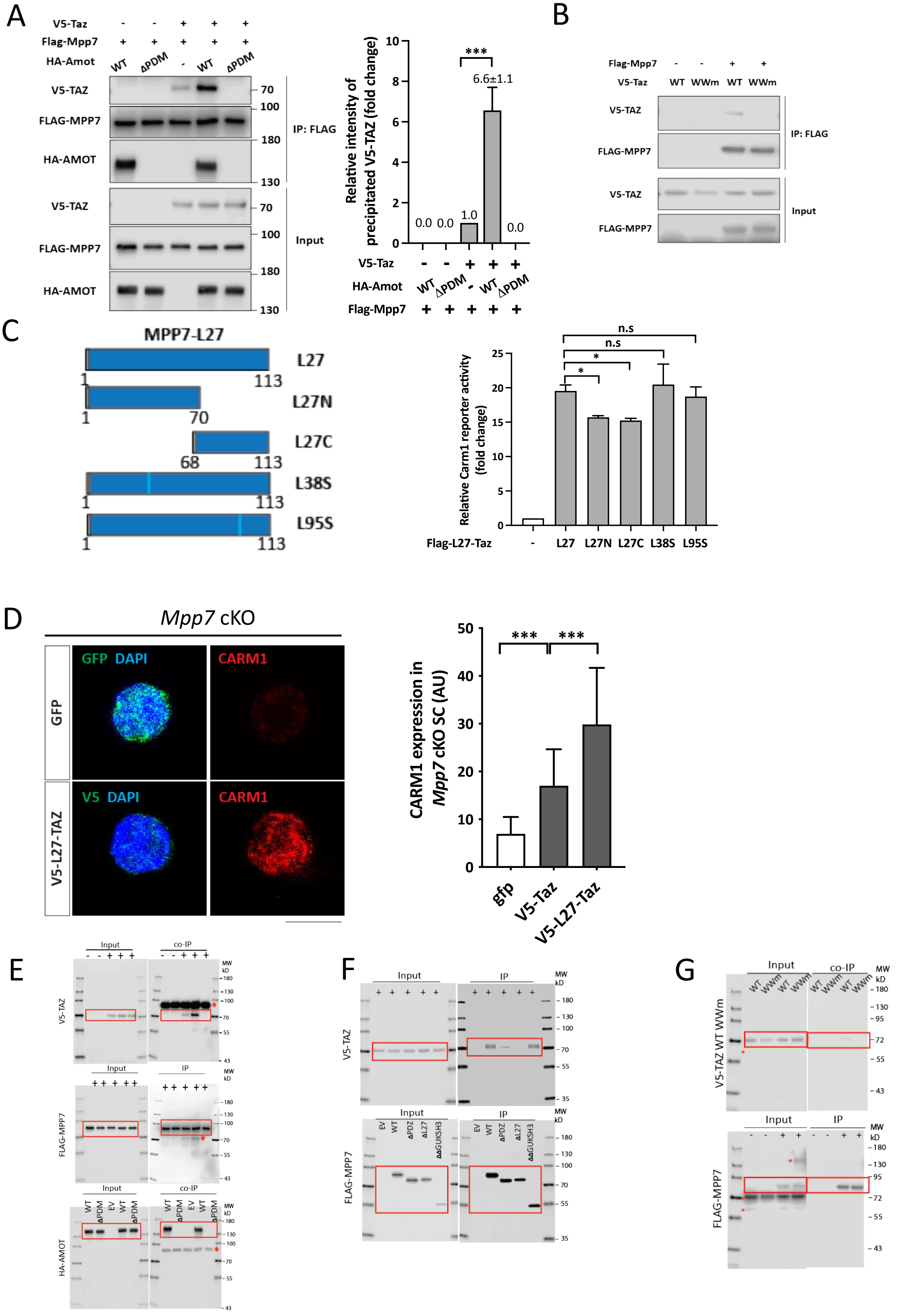
A
V5-Taz
Flag-Mpp7
HA-Amot
WT
ΔPDM
V5-TAZ
FLAG-MPP7
HA-AMOT
IP: FLAG
Input
70
100
180
130
Relative intensity of precipitated V5-TAZ (fold change)
***
6.6±1.1
1.0
0.0 0.0
0.0
B
Flag-Mpp7
V5-Taz
WT WWm WT WWm
V5-TAZ
FLAG-MPP7
IP: FLAG
Input
C
MPP7-L27
L27
L27N
L27C
L38S
L95S
1
113
70
68
Relative Carm1 reporter activity (fold change)
n.s
*
Flag-L27-Taz
D
Mpp7 cKO
GFP DAPI
CARM1
GFP
V5 DAPI
V5-L27-TAZ
CARM1 expression in Mpp7 cKO SC (AU)
***
gfp
V5-Taz
V5-L27-Taz
E
Input
co-IP
IP
V5-TAZ
FLAG-MPP7
HA-AMOT
MW kD
F
Input
IP
MW kD
V5-TAZ
FLAG-MPP7
EV
WT
ΔPDZ
ΔL27
ΔΔGUKSH3
G
Input
co-IP
IP
MW kD
V5-TAZ WT WWm
FLAG-MPP7
WT
WWm

Fig. S6

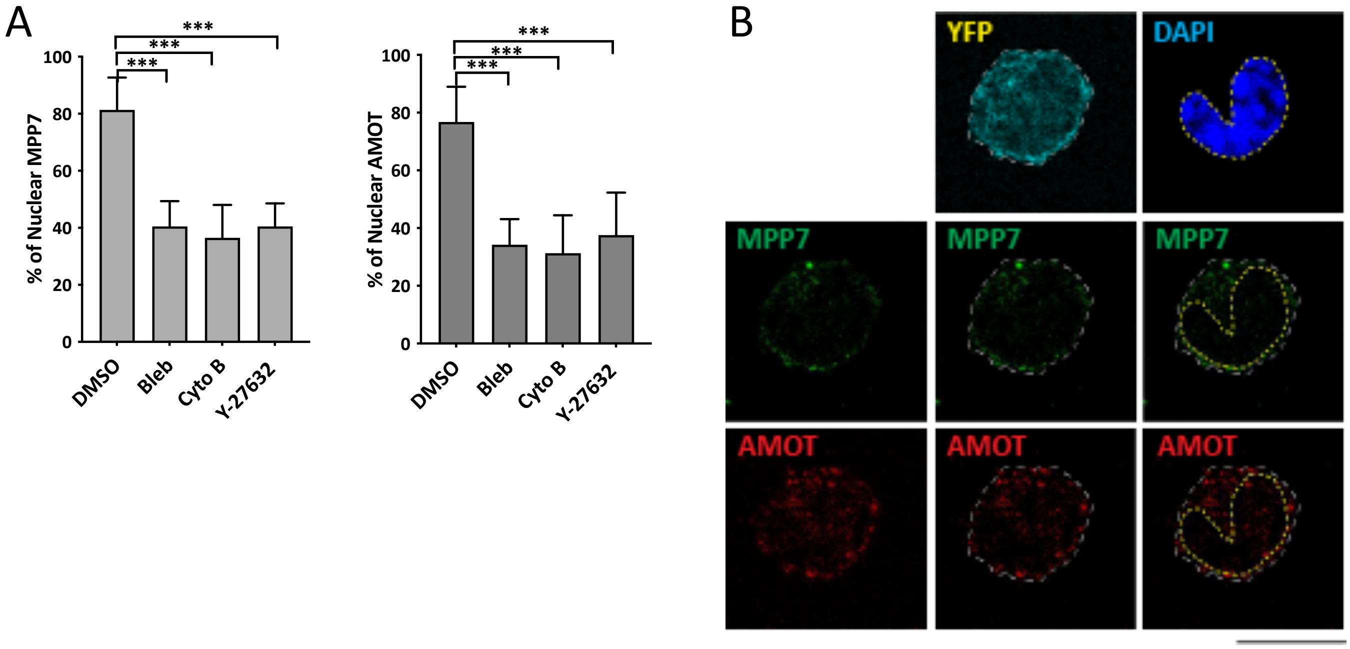
A
***
***
***
100
80
60
40
20
0
% of Nuclear MPP7
DMSO
Bleb
Cyto B
Y-27632
***
***
***
100
80
60
40
20
0
% of Nuclear AMOT
DMSO
Bleb
Cyto B
Y-27632
B
YFP
DAPI
MPP7
MPP7
MPP7
AMOT
AMOT
AMOT

Fig. S7

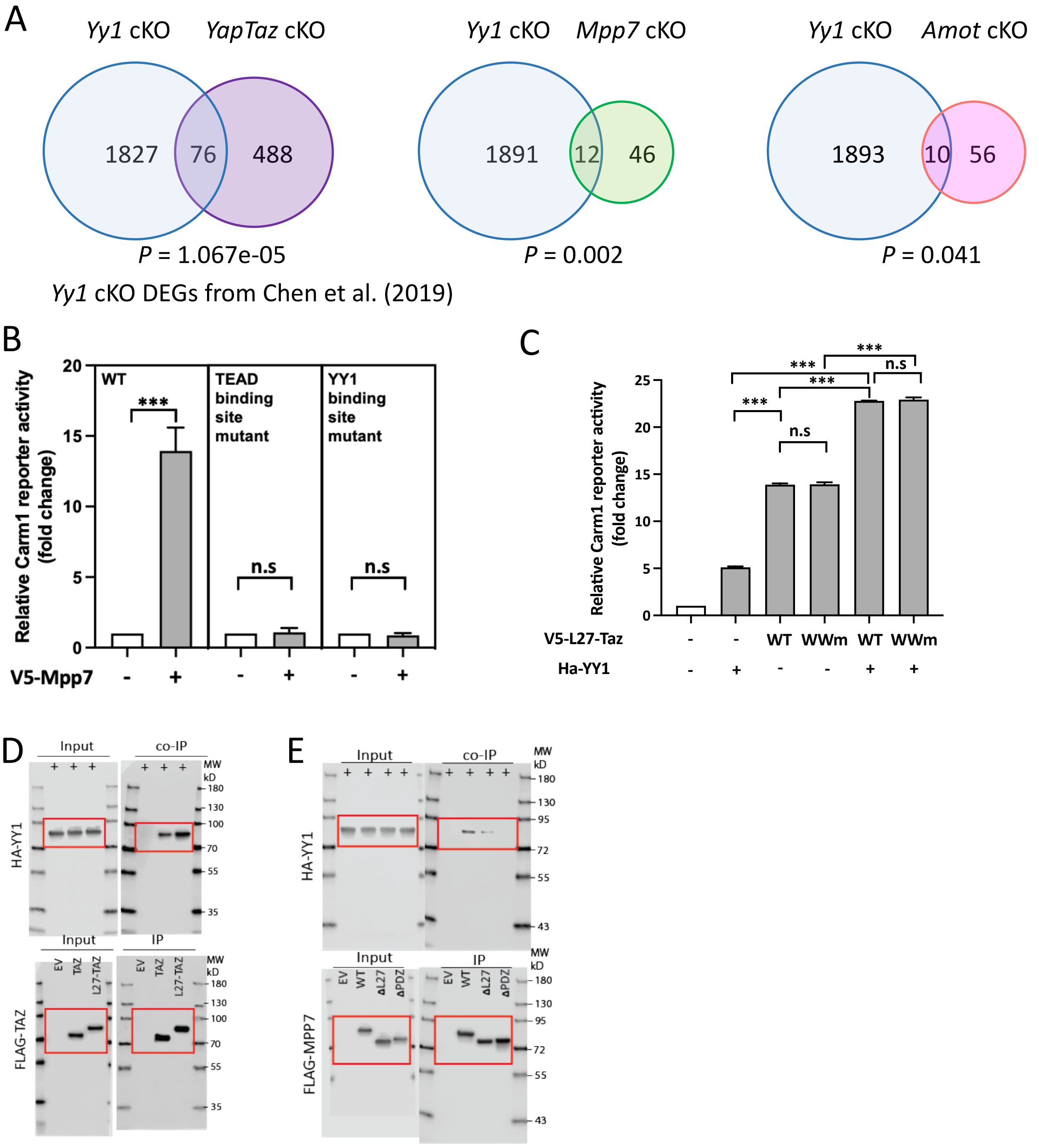
A
Yy1 cKO
YapTaz cKO
1827
76
488
P = 1.067e-05
Yy1 cKO
Mpp7 cKO
1891
12
46
P = 0.002
Yy1 cKO
Amot cKO
1893
10
56
P = 0.041
Yy1 cKO DEGs from Chen et al. (2019)
B
Relative Carm1 reporter activity (fold change)
WT
TEAD binding site mutant
YY1 binding site mutant
***
n.s
n.s
V5-Mpp7
C
Relative Carm1 reporter activity (fold change)
***
n.s
V5-L27-Taz
WT
WWm
Ha-YY1
D
Input
co-IP
IP
HA-YY1
FLAG-TAZ
EV
TAZ
L27-TAZ
MW kD
E
FLAG-MPP7
WT
ΔL27
ΔPDZ
